# Improvement of bone properties in children with osteogenesis imperfecta after pamidronate: a bone biopsy study

**DOI:** 10.1093/jbmrpl/ziae161

**Published:** 2024-12-11

**Authors:** Delphine Farlay, Mathilde Cornier, Sébastien Rizzo, Valérie Cormier-Daire, Maelle Charpié, Graziella Pinto, Hina Simonnet, Isabelle Badoud, Maude Gerbaix, Pascale Chavassieux, Roland Chapurlat

**Affiliations:** INSERM UMR 1033, Univ Lyon, Université Claude Bernard Lyon 1, F-69008 Lyon, France; INSERM UMR 1033, Univ Lyon, Université Claude Bernard Lyon 1, F-69008 Lyon, France; INSERM UMR 1033, Univ Lyon, Université Claude Bernard Lyon 1, F-69008 Lyon, France; Department of Clinical Genetics and Reference Centre for Constitutional Bone Diseases, INSERM U1163, Université de Paris, F-75015 Paris, France; Department of Clinical Genetics and Reference Centre for Constitutional Bone Diseases, INSERM U1163, Université de Paris, F-75015 Paris, France; Department of Pediatric Endocrinology, Necker-Enfants Malades Hospital, F-75015 Paris, France; Service de Médecine Physique et de Réadaptation pédiatrique Hôpital Armand-Trousseau, F-75012 Paris, France; Division of Bone Diseases, Department of Internal Medicine Specialties, Geneva University Hospital, CH-1211 Geneva, Switzerland; Division of Bone Diseases, Department of Internal Medicine Specialties, Geneva University Hospital, CH-1211 Geneva, Switzerland; INSERM UMR 1033, Univ Lyon, Université Claude Bernard Lyon 1, F-69008 Lyon, France; INSERM UMR 1033, Univ Lyon, Université Claude Bernard Lyon 1, F-69008 Lyon, France

**Keywords:** OI, pamidronate, mineral/collagen properties, bone mineralization, nanoindentation

## Abstract

OI, or bone brittle disease, is characterized by increased mineralization of bone matrix independently of clinical severity. So, a beneficial effect of antiresorptive treatments such as bisphosphonates (BP) is questionable. We aim to compare the bone matrix characteristics before and after BP pamidronate (PAM). Fifty-eight children (9 ± 5 yr-old) with OI (Type I, III, IV, V, VI, XI, or unknown) received intravenous PAM for 2 yr and underwent transiliac bone biopsies before (*n* = 57) and after (*n* = 35) treatment. Compared with age-matched controls, untreated OI was characterized by cortical and cancellous rarefaction. Two years of PAM in OI patients significantly decreased bone remodeling activity, increased cortical thickness, improved the maturation of both organic and mineral matrix, and most of the nanomechanical properties, despite further increase in the degree of mineralization. Overall, in addition to a gain in bone mass, our results showed for the first time that PAM is able to increase the maturation of mineral crystals and collagen matrix contributing to its antifracture efficacy in OI patients.

## Graphical Abstract

**Graphical Abstract:**
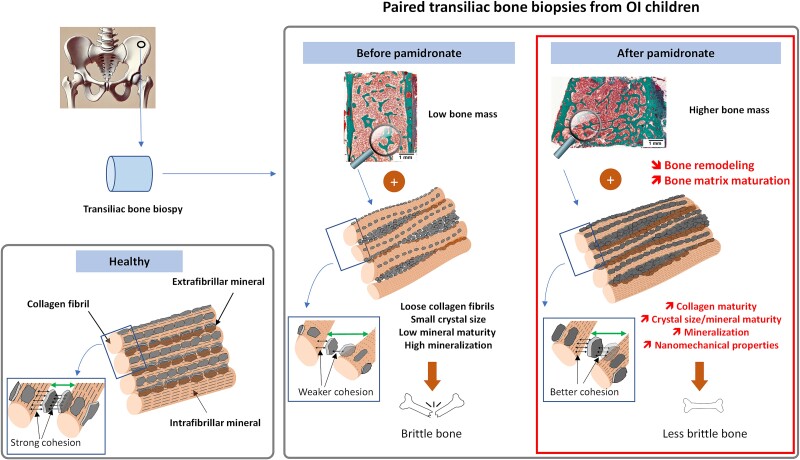

## Introduction

OI is a family of rare genetic diseases of connective tissue, mainly of autosomal dominant transmission, characterized by bone fragility, sarcopenia, skeletal deformities, and other nonskeletal findings such as short stature, hearing loss, dental abnormalities, blue sclera, and cardiovascular abnormalities. The prevalence ranges from 1: 15 000 to 1:20 000.^1^ The OI classification proposed by Sillence in 1979 was based on the phenotype and identified 4 types from mild to lethal form.^2^ So far, more than 22 types of OI have been described, mainly related to an autosomal dominant mutation of COL1A1 and COL1A2 (80%), but autosomal recessive mutations of several other genes have been reported. They are related not only to the production, folding, stability, and processing of type 1 collagen but also to genes involved in the bone matrix mineralization and osteoblast function such as SERPINF1, SPARC, CRTAP, P4HB, and SERPINH1.^3^ These mutations result in either a decreased amount of collagen or an abnormal collagen (aberrant collagen structure, glycine mutation) associated with modified amounts of bone non collagenous proteins.^4^

Bone is a composite material in which 2 phases (organic and mineral) combine to produce mechanical properties superior to those of each phase to achieve remarkable material properties, including both stiffness and toughness to maintain optimal bone strength. Load is shared between collagen matrix and mineral crystallites, and this involves load transfer from the collagen matrix to mineral. Thus, bone material properties depend not only on the properties of each phase but also on the properties of the interface between the 2 phases. OI bone is characterized by an increased brittleness. In an OI mice model (oim), the high bone brittleness was attributed to an abnormal interaction of the mineral phase with the collagenous matrix. Indeed, while the majority of mineralized femora mechanical properties were lower in oim than in WT mice, the tensile measurements (ultimate strain) of demineralized bones revealed no differences between both groups, suggesting that the organic matrix itself was not brittle.^5^

In addition, human and mice studies showed that the OI bone is characterized by elevated mineralization of the bone matrix independently of the clinical severity of OI,^6–11^ also found in OI Type VI with mineralization defect and evidenced by Fratzl-Zelman.^12^ In OI bone, this “hypermineralization” does not mechanically contribute to matrix elastic properties.^13^ Moreover, other mice studies pointed out the low toughness of OI bone.^14^^,^^15^ The cause for the high mineralization in OI is unknown but some mechanisms have been proposed such as the over hydroxylation and glycosylation in the type I collagen triple helical domain,^3^^,^^16^^,^^17^ the presence of smaller collagen fibrils, and increased space between the molecules allowing a larger space for mineral deposition.^18^ Moreover, several studies showed that mineral crystals were smaller in OI than in normal bone, with abnormal location, alignment, and orientation of mineral crystals with respect to the collagen fibrils.^11^^,^^13^^,^^19^^,^^20^ Mineral crystals alignment in oim/oim mice is uniform in all orientations, whereas it is preferentially oriented in the longitudinal direction in normal bone.^19^^,^^21^ This underlines the importance of the organic matrix for the process of mineralization (correct arrangement of collagen network, amount, and functionality of bone non-collagenous proteins), as the organic matrix serves as a primary template for crystal orientation and mineralization. Therefore, the association of high mineralization, low biomechanical competence of the collagenous matrix, and low bone mass explain the severe bone brittleness in OI. Besides, the degree of mineralization (DMB), the characteristics of the mineral (crystal size/perfection, maturity), and collagen such as the collagen maturity play a major role in the bone strength.

One of the most commonly used treatments in children with OI are intravenous bisphosphonates. Bisphosphonates have been shown to decrease bone remodeling activity and increase the bone DMB in postmenopausal osteoporosis that mainly contribute its prolonged antifracture efficacy.^22^ Pamidronate (PAM) has been the most widely used bisphosphonate in OI in the 1990s and 2000s. Today, intravenous zoledronic acid has largely replaced the use of PAM in such patients. However, PAM and zoledronic acid are both aminobisphosphonates, so studying the impact of PAM on material properties could be helpful to better understand the mechanism of action of aminobisphosphonates in OI and consequently the mechanisms leading to a reduction in bone fragility in OI that remain poorly understood. Indeed, excessive mineralization of bone matrix in a bone already overmineralized should contribute to increase the brittleness, but it is not the case, as bisphosphonates are effective in reducing fracture incidence and reducing pain in OI children^23^. In 14 pairs of bone biopsies from children with OI, no change in mineralization assessed by quantitative electron backscattered imaging (qBEI) was found, and no change in material properties measured by nanoindentation on a subset of 6 pairs were found after 24 mo of PAM.^24^ However, as PAM reduces bone remodeling activity, a higher bone mineralization would be expected. Thus, we aim to investigate, on a large number of paired iliac crest biopsies of children suffering from OI, the effect of 24 mo of PAM on bone mineralization, the characteristics of organic and mineral matrix, and the nanomechanical properties.

## Materials and methods

### Patients

Fifty-eight patients diagnosed with OI including 35 boys and 23 girls (mean age: 9 ± 5 yr; range: 2-22 yr) have been recruited between 1999 and 2004, at Necker (*n* = 18) and Trousseau hospitals (*n* = 40), Paris, France. Twenty-nine of these children have been included in the previous study evaluating the effect of 1 yr of PAM on the fracture rate.^23^ Twenty-one children were diagnosed as having type I OI (COL1A1 or COL1A2 mutations), 11 had type III OI (1 with CCDC134 mutation), 16 had type IV OI (COL1A1, COL1A2 mutations), 1 had type V (IFITM5 mutation), 2 had type VI (Serpin F1 mutation), 1 had type XI (FKBP10 mutation), and 6 had undefined types of OI. PAM was administered intravenously in cycles of 3 consecutive days at the dose of 0.5 mg/kg/d given every 2 or 3 mo in infants (<2 yr-old), and 0.5 mg/kg the first day of the first infusion cycle, followed by 1 mg/kg/d thereafter given at 4-mo intervals in children older than 2 yr. Overall, the cumulative dose was 9 and 8.5 mg/kg/yr in infants and children, respectively. All patients received a daily supplementation of 1200 IU of vitamin D. The dose of vitamin D was then periodically adjusted in order to avoid hypercalciuria and nephrocalcinosis. Since this study corresponds to a retrospective case series in which bone biopsies were performed between 1999 and 2004 in order to assess the effect of PAM on bone structure and remodeling by histomorphometry, it did not require any local ethics committee evaluation.

### Transiliac bone biopsies

Transiliac bone biopsies were performed in children before and after 24 mo of PAM treatment. For the second biopsy, contralateral side was collected. Before each biopsy, patients received a double tetracycline labeling as follows: 2 sets, the first set before the initiation of the treatment (baseline labeling) with two 2-d cycles of 15-20 mg/kg per day demeclocycline, 10 d apart, and the second set (same that the first one). For this study, 92 bone biopsies have been performed, 57 patients had a bone biopsy before the treatment, and 35 had a second bone biopsy after the treatment (except 1 without bone biopsy before, but only after PAM). Among the children having underwent bone biopsies, 34 (21 boys, 13 girls) have had one before and one after treatment (paired biopsies). In this study, only the results obtained on paired bone biopsies (*n* = 68) from children treated with PAM are presented, as well as the data at baseline for OI type V (*n* = 1), type VI (*n* = 2), and XI (*n* = 1). All paired bone biopsies have been analyzed for bone matrix characterization but some samples, only cortical bone or cancellous bone was preserved and could be assessed. Due to the poor quality of some biopsies only 11 pairs were assessed by histomorphometry. Bone biopsies were collected over 5 yr; however, histomorphometry measurements were done over the years by a same observer. Digitized quantitative X-ray microradiography, Fourier transform infrared microspectroscopy (FTIRM), microindentation, and nanoindentation were performed by batch each one by a same observer.

### Bone histomorphometry

After fixation in 70% ethanol and dehydration in 100% ethanol, specimens were embedded in methylmethacrylate. Three sets of 8 μm-thick sections were cut with a microtome Polycut E (Reichert-Jung, Leica, Germany), 200 μm apart, in the central zone of the sample. Sections were stained with Goldner trichrome, solochrome cyanin R, and some sections were left unstained for the measurement of the tetracycline labels under fluorescence.

The quantitative analysis was performed in unbroken samples with sufficient cancellous bone area. The abbreviations of the bone histomorphometic parameters used were those recommended by the ASBMR Histomorphometric Nomenclature Committee.^25^ The parameters of bone structure were measured with an automatic image analyzer (Bone V3.5, Explora Nova®, La Rochelle, France). The static parameters reflecting resorption and formation, and the dynamic parameters of bone formation and mineralization were measured using a semiautomatic image analyzer (Tablet’Measure V1.54, Explora Nova®, La Rochelle, France). Structural parameters included cortical (Ct) thickness (Ct.Th, μm), porosity (Ct.Po, %), and cancellous (Cn) bone volume (Cn-BV/TV, %). The parameters of microarchitecture, trabecular thickness (Tb.Th, μm), number (Tb.N, #/mm), and separation (Tb.Sp, μm) were derived from area and perimeter measurements according to Parfitt’s formulae.^26^ Bone resorption was assessed with measurements of eroded surface (ES/BS, %) and the number of osteoclasts per bone surface (Oc.N/BS; #/mm). Static bone formation was reflected by osteoid surface (OS/BS, %), volume (OV/BV, %), thickness (O.Th, μm), and osteoblastic surface (Ob.S/BS, %). All of these parameters were measured on Goldner-stained sections. Mean wall thickness (W.Th, μm) was measured on solochrome cyanin R-stained sections under polarized light. The mineral apposition rate (MAR, μm/d) and ratio of mineralizing surface to bone surface (MS/BS, % calculated as double plus half of single-labeled surfaces) were analyzed on unstained sections under ultraviolet light. Bone formation rate (BFR/BS, μm^3^/μm^2^/d; =[MS/BS] × MAR), adjusted apposition rate (Aj.AR, μm/d; =BFR/OS), formation period (FP, d; =W.Th/Aj.AR), active formation period (FPa+, d; =W.Th/MAR), mineralization lag time (Mlt, d; = O.Th/Aj.AR), and activation frequency (Ac.f, per year; =[BFR/BS]/W.Th x 365) were calculated.

### Digitized quantitative X-ray microradiography

The DMB of bone (g/cm^3^) was assessed by digitized quantitative microradiography, according to the method previously described.^27^ Briefly, 100 ± 1 μm thick-sections were analyzed by digitized X-ray microradiography, under high voltage 40 kV, current 50 μA and power of 2 W. A Photonic science CCD camera FDI VHR 11 M was used to detect the signal. The image digitization step was done with a 12-bit digital image detector (pixel size: 9 μm, object pixel size: 0.83 μm). An aluminum step-wedge reference and gray level of each step were converted in g mineral/cm^3^. using code from MATLAB program (MathWorks, Natick, MA, United States). A threshold of 0.6 g/cm^3^ was used. The mean DMB and mean HI (HI = full-width at half-maximum of the curve of distribution) of the DMB were expressed in g mineral/cm^3^ and measured in cancellous and cortical bone separately.

### Fourier transform infrared microspectroscopy

Briefly, 2 μm-thick sections were cut with a microtome and FTIRM analysis was performed in transmission mode on a Spectrum 100 spectrometer equipped with an autoImage microscope (Perkin-Elmer, Norwalk, CT, United States). Acquisition was done on a 50 x 50 μm bone area, with a 2 cm^-1^ spectral resolution. Thirty bone areas per sample were scanned when possible (depending on the size of the sample, or on the presence of both cortical and trabecular bone), with 10 measures in each cortical and 10 measures in trabecular bone. Curve fitting of spectra was performed automatically using a custom software.^28^ The mineral to organic ratio (min/org 1184-910 cm^−1^/1712-1592 cm^−1^), the mineral crystallinity (reflecting both crystal size and perfection) as: cryst = 1/full width at half-maximum of the 604 cm^−1^ peak (apatitic phosphate environment), the mineral maturity (min mat) which is calculated as the area ratio of the apatitic phosphate over non apatitic phosphate (1030/1110 cm^−1^ area ratio) and reflects the age of mineral, and the collagen maturity (coll mat), which is calculated as the ratio of organic matrix bands (1660/1690 cm^−1^ area ratio). Results are expressed as mean ± standard deviation (SD) for each bone envelope (cortical or cancellous).

### Microindentation

The microhardness of bone was assessed using a Vickers indenter (Micromet 5104; Buehler, Lake Bluff, IL, United States), with a test load of 25 g applied during 10 s, on the remaining block, after polishing with alumina suspension. Thirty indents were performed by sample, with 10 indents in each cortical (20 in both cortical) and 10 indents in cancellous bone when possible. Microindentation was performed in randomly selected areas of the bone surface, separated by at least 500 μm over the whole bone tissue area. The size of the impressions was measured from the direct measurement of diagonal dimensions using manufacturer software (Omnimet HMS v.2.31; Buehler), and the microhardness (Hv) was derived using the following formulae Hv = 1854.4 P/ d2 (where Hv is Vickers microhardness expressed in kg/mm^2^, P is test load in kg, and d is the mean length of the 2 diagonals expressed in mm). Results are expressed as mean ± SD for each bone envelope (cortical or cancellous).

### Nanoindentation

As nanoindentation is time-consuming, it was performed in only 10 paired biopsies from OI children (4 OI Type I, 2 OI Type III, 4 OI Type IV) and 1 OI type VI. Measurements were done in longitudinal direction, at the bone structural unit (BSU) level under wet condition, on selected OI bone biopsies for which the cutting axis was perpendicular to the Haversian canals. Nano-indentation allows for assessment of the material level properties of bone tissue. Nano-hardness tester (NHT, CSM Instruments, Peseux, Switzerland) was used, and the protocol was described in a previous work.^29^ The indentation modulus is derived from the known properties of the indenter tip and the reduced modulus, representing the sum of compliance of the material and the diamond indenter. The Indentation modulus combines with the local elastic modulus and Poisson ratio of the specimen. Hardness is defined by an applied force P divided by contact area Ac. The indents were set to a 900 nm depth with an approximate speed of 76 mN/min for both loading and unloading, and at maximum load, a 10 s holding period was applied. Finally, the limit of the maximal allowable thermal drift was set to 0.1 nm/s. Indentation modulus (GPa), hardness (MPa) and dissipated energy (pJ) of the bone were determined. Ten indents were performed in the cortical bone (5 in osteonal and 5 in interstitial tissues) and 10 indents were also performed in trabecular bone (osteonal and interstitial tissues). Osteonal bone was defined as recent formed “packets” in cancellous bone, localized at the surface of the trabeculae in contact with the bone marrow. Interstitial bone was localized at the center of the trabeculae or in cancellous nodes.

### Statistical analysis

Results are expressed as mean *+* SEM. At baseline, comparisons between the different types of OI were performed with Kruskall-Wallis test and the comparison between 2 groups was performed with Mann-Whitney tests. Only OI type I, III and IV were tested, and OI type V, VI and XI were not tested due to the too small number of biopsies per group (*n* = 1 or *n* = 2). To compare histomorphometric measurements in untreated OI patients with healthy children, individual data from each patient was age-matched with the mean control value previously published.^30^ To test the effect of PAM treatment, comparison between paired values (before/after) was performed by Wilcoxon signed rank test using SPSS v20 (SPSS Inc, Chicago, IL, United States).

## Results

Fifty-seven biopsies have been collected before treatment and qualitatively analyzed but due to the small size and/or the quality of the samples, 31 were measurable by bone histomorphometry (13 OI type I, 7 OI type III, 9 OI type IV, 1 OI type VI, 1 OI type XI). The bone material properties were assessed in 34 biopsies (16 OI type I, 6 OI type III, 10 OI type IV, 1 type VI, 1 unknown). For nanoindentation, 4 OI type I, 2 OI type III, 4 OI type IV, and 1 OI type VI were selected for analysis.

Results at baseline for the different types of OI are shown in Figures S1-S3. Bone histomorphometry (Figure S1) showed that Ct.Th was significantly lower in OI type IV than in OI type I (*p*=.013). In OI type III, OS/BS and FP were significantly higher than in OI type I (*p*=.024 and *p*=.018 respectively).

No significant differences were found for the DMB, heterogeneity index and Vickers microhardness between the different types of OI (Figure S2). FTIRM analysis showed that Ct and Cn crystallinity, and Ct mineral maturity were significantly lower in OI type IV than in OI type I (*p*=.002, *p*=.002 and *p*=.0007, respectively). In OI type III, only Ct mineral maturity was significantly lower than in OI type I (*p*=.015, Figure S2). For nanoindentation, Cn Old-indentation modulus, -hardness and -dissipated energy were significantly lower in OI Type IV compared with OI type I (*p*=.021, *p*=.043, *p*=.021, respectively, Figure S3).

### Untreated OI compared with healthy controls

#### Bone histomorphometry

**Figure 1:**
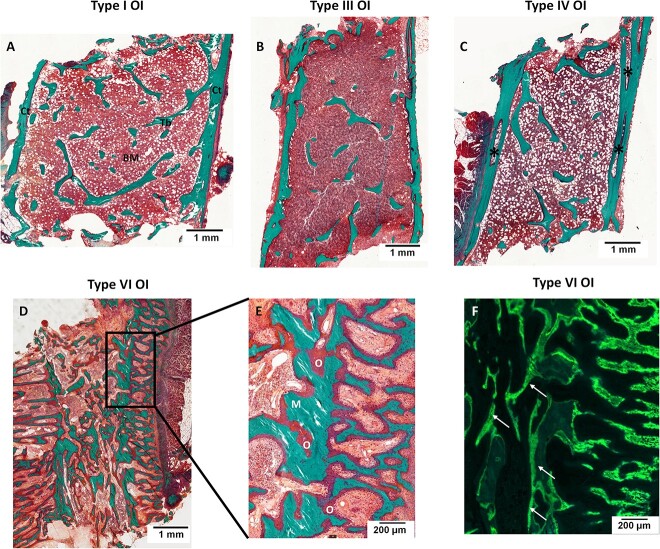
Histological sections of iliac bone in a 4-yr-old boy with type I OI (A), a 3-yr-old girl with type III OI (B), a 3-yr-old girl with type IV OI (C), and a 2-yr-old boy with type VI OI (D) showing a cortical (Ct) and trabecular (Tb) bone rarefaction with a loss of the trabecular connectivity: Goldner stained sections showing the mineralized bone (M) in green, the osteoid (O), and cells in the bone marrow (BM) in red. Note the thin cortical thickness in the 3 types of OI (A-C) and the important cortical porosity in type IV OI (black stars, C). Type VI OI characterized by a marked mineralization defect as shown by the extended osteoid (O) surface in red, the thickening of osteoid seams (D, E), and the diffuse tetracycline labels showed by white arrows (F, unstained section under ultraviolet light).

When compared with age-matched healthy children, OI was characterized by a marked trabecular and cortical bone rarefaction (Cn BV/TV (%), *p*<.0001; Ct.Th, *p*=.0002 respectively) with a decreased trabecular number (*p*<.0001) (Table 1, Figure 1A-D). This bone rarefaction resulted from a significant diminution of W.Th (*p*<.0001) which reflected the amount of bone formed at the bone structural unit (Table 1).

**Table 1:** Histomorphometric parameters in OI children before PAM treatment. Comparison with age-matched control.

	**Controls** ^a^ **(*n* = 31)**	**Pre-treatment OI** **(*n* = 31)**	***p*** ^c^
**Ct.Th (μm)**	879.0 (28.1)^b^	585.5 (52.0)	0.0002
**Cn-BV/TV (%)**	21.9 (0.6)	11.5 (0.9)	<0.0001
**Tb.Th (μm)**	128.7 (4.1)	116.2 (6.2)	0.08
**Tb.Sp (μm)**	601.2 (137.6)	1213.8 (169.8)	<0.0001
**Tb.N (/mm** ^**2**^ **)**	1.7 (0.01)	1.0 (0.1)	<0.0001
**Cn-W.Th (μm)**	40.1 (0.9)	24.9 (1.1)	<0.0001
**Cn-ES/BS (%)**	15.7 (0.2)	12.8 (1.3)	0.06
**Cn-Oc.N/BS (#/mm** ^**2**^ **)**	0.9 (0.1)	0.27 (0.03)	<0.0001
**Cn-OS/BS (%)**	27.8 (1.0)	36.3 (2.6)	0.008
**Cn-OV/BV (%)**	2.9 (0.2)	7.7 (1.3)	<0.0001
**Cn-O.Th (μm)**	6.2 (0.1)	12.9 (0.7)	<0.0001
**Cn-Ob.S/BS**	7.7 (0.2)	19.1 (1.9)	<0.0001
**Cn-MAR (μm/d)**	0.93 (0.02)	1.00 (0.03)	0.03
**Cn-MS/BS (%)**	12.6 (0.3)	13.6 (1.2)	0.61
**Cn-BFR (μm** ^**2**^ **/μm/d)**	0.118 (0.004)	0.140 (0.014)	0.23
**Cn-Aj.AR (μm/d)**	0.436 (0.006)	0.441 (0.045)	0.98
**Ac.f (/yr)**	1.093 (0.054)	2.097 (0.243)	0.0002
**FP (d)**	104.4 (0.9)	93.7 (19.0)	0.07
**FPa + (d)**	44.1 (1.2)	25.0 (1.2)	<0.0001
**Mlt (d)**	15.4 (0.2)	52.9 (12.3)	<0.0001

a Age-matched control values from Glorieux et al.^30^;

b mean (SEM).

c Wilcoxon signed rank test. Abbreviations: Ct, cortical; Cn, cancellous; BV, bone volume; Tb, trabecular; Th, thickness; N, number; Sp, separation; W.Th, wall thickness; ES, eroded surface; Oc.S, osteoclast surface, Oc.N, osteoclast number; OS, osteoid surface; OV, osteoid volume; Ob.S, osteoblast surface; MAR, mineral apposition rate; MS, mineralizing surface; BFR/BS, bone formation rate; Aj.AR, adjusted apposition rate; Ac.f, activation frequency; FP, formation period; FPa+, active formation period; Mlt, mineralization lag time; PAM, pamidronate.

The qualitative observations showed a bone texture mainly lamellar except in 6 samples with the presence of woven bone, 4 of them being in children younger than 6 yr. Signs of marked mineralization defects were observed in 3 cases, 2 of which were type VI OI (Figure 1D-F).

The decrease in W.Th explained the apparent increase in Ac.f, the level of bone turnover being normal as shown by MS/BS and BFR/BS. The duration of the formation period decreased with a marked reduction of the active formation period (*p*<.0001). There was no mineralization defect despite the increases in osteoid parameters which could be related to a significant extension of Mlt but the mineralization rate was normal except in the 2 cases of type VI OI which showed a severe mineralization defect (Figure 1D and E).

### Effect of 24 mo of PAM

#### Bone histomorphometry

After 24 mo of PAM, 35 bone biopsies were obtained, 21 (10 OI type I, 2 OI type III and 9 OI type IV) were measurable by histomorphometry but an evaluable pair of a pre- and post-treatment biopsies was obtained in only 11 patients (7 OI type I, 4 OI type IV).

**Figure 2:**
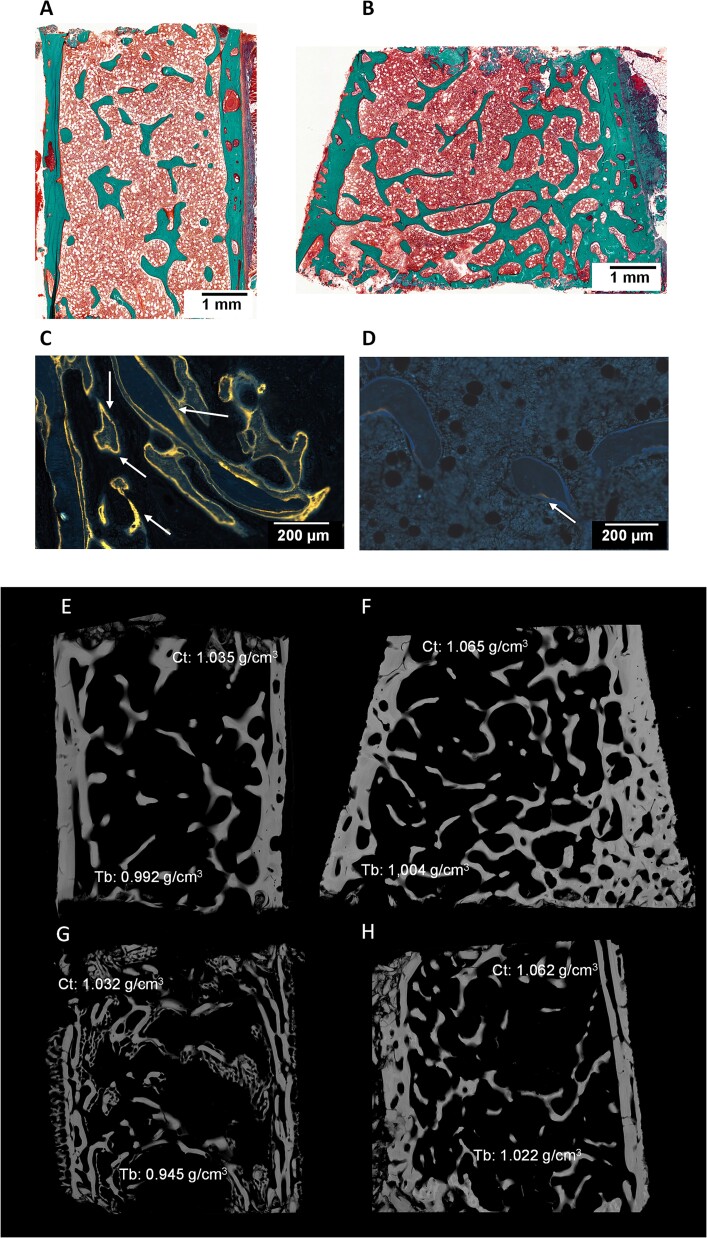
Goldner stained sections of iliac bone in a 8-yr-old girl with type I OI before (A) and after 2 yr of PAM (B) showing an increased cortical and cancellous bone mass with connected trabeculae. Unstained sections of iliac bone in a 3-yr-old boy with type I OI: The extended labeled surfaces (white arrows) before treatment (C) are markedly reduced after 2 yr of pamidronate (D). X-rays digitized microradiographies assessed by pairs before (E-G) and after (F-H) 24 mo of pamidronate (same patients than in A-D). Note the higher gray levels illustrating the higher degree of mineralization of bone (DMB, g/cm^3^) after treatment.

Compared with baseline, 24 mo of PAM significantly increased Ct.Th (*p*<.005), and tended to increase Cn-BV/TV (*p*=.1). The marked decreases in MS/BS, BFR/BS and Ac.f (0.01<*p*<.02) revealed a decrease in bone turnover after PAM treatment and the consequent diminution in BFR. However, the duration of the active bone formation and the amount of bone formed at the BSU level remained unchanged (Figure 2A-D, Table 2).

**Table 2:** Histomorphometric parameters in OI children before and after 2 yr of PAM treatment.

	**OI (*n* = 11)**		***p*** ^b^
	**Pre-treatment**	**Post-treatment**	
**Ct.Th(μm)**	481.4 (58.3)^a^	734.4 (83.8)	0.005
**Ct.Po (%)**	5.97 (1.4)	7.2 (1.2)	0.3
**Cn-BV/TV (%)**	12.5 (0.9)	18.8 (4.2)	0.1
**Tb.Th (μm)**	115.8 (5.4)	124.3 (13.8)	0.8
**Tb.Sp (μm)**	862.5 (96.3)	682.3 (98.4)	0.07
**Tb.N (/mm** ^**2**^ **)**	1.1 (0.1)	1.5 (0.2)	0.1
**Cn-W.Th (μm)**	23.9 (2.2)	25.0 (1.5)	0.5
**Cn-ES/BS (%)**	10.2 (1.8)	10.0 (1.7)	0.9
**Cn-Oc.N/BS (#/mm** ^**2**^ **)**	0.3 (0.05)	0.3 (0.05)	0.9
**Cn-OS/BS (%)**	33.3 (4.6)	29.4 (3.5)	0.7
**Cn-OV/BV (%)**	5.9 (1.3)	3.3 (0.5)	0.2
**Cn-O.Th (μm)**	13.5 (1.8)	12.7 (0.5)	0.9
**Cn-MAR (μm/d)**	0.99 (0.05)	0.89 (0.02)	0.03
**Cn-MS/BS (%)**	16.1 (2.5)	5.3 (0.9)	0.02
**Cn-BFR (μm** ^**2**^ **/μm/d)**	0.165 (0.027)	0.069 (0.021)	0.02
**Cn-Aj.AR (μm/d)**	0.503 (0.063)	0.173 (0.029)	0.005
**Ac.f (/yr)**	2.650 (0.498)	0.746 (0.159)	0.01
**FP (d)**	60.2 (15.0)	209.3 (54.5)	0.02
**FPa + (d)**	24.8 (2.5)	28.4 (2.3)	0.3
**Mlt (d)**	31.8 (5.7)	109.6 (36.3)	0.01

a mean (SEM).

b Wilcoxon signed rank test. Abbreviations: Ct, cortical; Cn, cancellous; Po, porosity; BV, bone volume; Tb, trabecular; Th, thickness; N, number Sp, separation; W.Th, wall thickness; ES, eroded surface; Oc.N, osteoclast number; OS, osteoid surface; OV, osteoid volume; Ob.S, osteoblast surface; MAR, mineral apposition rate; MS, mineralizing surface; BFR/BS, bone formation rate; Aj.AR, adjusted apposition rate; Ac.f, activation frequency; FP, formation period; FPa+, active formation period; Mlt, mineralization lag time; PAM, pamidronate.

**Figure 3:**
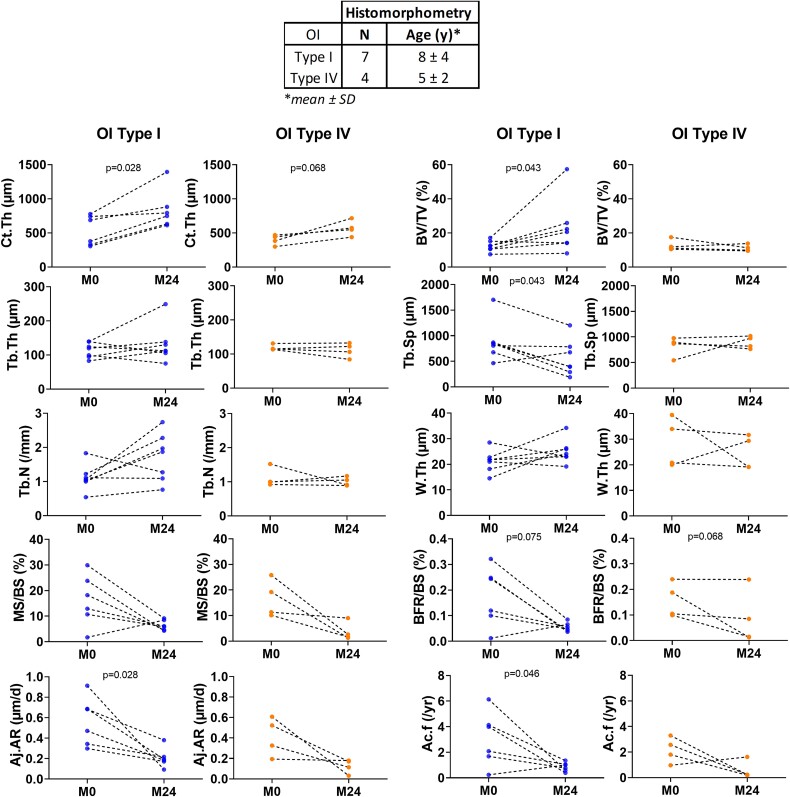
Evolution of histological parameters after 2 yr of PAM in OI type I (blue) and OI type IV (orange). Abbreviations: Ct, cortical; Cn, cancellous; BV, bone volume; Tb, trabecular; Th, thickness; N, number; PAM, pamidronate; Sp, separation; W.Th, wall thickness; W.Th, mean wall thickness; MS, mineralizing surface; BFR/BS, bone formation rate; Aj.AR, adjusted apposition rate; Ac.f, activation frequency; p, Wilcoxon signed rank test.

When the results were separately analyzed by types of OI (by pairs, OI type I and IV), Ct.Th and Cn-BV/TV significantly increased after PAM (*p*=.028, *p*=.043 respectively) in OI type I, whereas Tb.Sp, Aj.Ar, Ac.f significantly decreased (*p*=.043, *p*=.028 and *p*=.046 respectively). No significant differences were found in OI type IV (Figure 3).

#### DMB of bone and heterogeneity index

The DMB of bone was analyzed in 34 paired bone biopsies. Compared with baseline, 24 mo of PAM increased the DMB in both cortical (*p*=.005) and cancellous (*p*=.013) bone without change in heterogeneity index (Table 3). This is illustrated by the higher gray levels after 24 mo of PAM on microradiographic images (Figure 2E-H).

**Table 3:** Degree of mineralization of bone (DMB), FTIRM parameters, heterogeneity index (HI), and microhardness (Hv) of iliac crests from children with OI before and after PAM treatment (*n* = 34)**.**

	**Pre-treatment OI** **(*n* = 34)**	**Post-treatment OI (*n* = 34)**	***p*** ^**a**^
**Ct DMB (g/cm** ^**3**^ **)**	1.027 (0.010)	1.065 (0.009)	0.005
**Ct HI (g/cm** ^**3**^ **)**	0.141 (0.004)	0.140 (0.004)	NS
**Cn DMB (g/cm** ^**3**^ **)**	0.971 (0.008)	1.000 (0.007)	0.013
**Cn HI (g/cm** ^**3**^ **)**	0.185 (0.012)	0.164 (0.010)	NS
**Ct Hv (kg/mm** ^**2**^ **)**	55.36 (1.14)	54.30 (1.05)	NS
**Cn Hv** (**kg/mm**^**2**^**)**	55.12 (1.39)	57.84 (1.30)	0.0009
**Ct Mineral maturity**	1.556 (0.031)	1.606 (0.025)	0.043
**Ct Crystallinity (cm** ^**-1**^ **)**	0.0343 (0.0003)	0.0352 (0.0002)	0.019
**Ct Mineral/matrix**	5.57 (0.24)	5.72 (0.17)	NS
**Ct Collagen maturity**	3.42 (0.13)	3.70 (0.09)	NS
**Cn Mineral maturity**	1.450 (0.025)	1.523 (0.025)	0.003
**Cn Crystallinity (cm** ^**-1**^ **)**	0.0345 (0.0003)	0.0352 (0.0003)	0.006
**Cn Mineral/matrix**	5.29 (0.19)	5.73 (0.16)	0.005
**Cn Collagen maturity**	3.30 (0.13)	3.63 (0.11)	0.014
	**Pre-treatment OI (*n* = 10)**	**Post-treatment OI (*n* = 10)**	
	**Osteonal**		
**Ct Indentation modulus (Gpa)**	5.31 (0.43)	7.49 (0.46)	0.0051
**Ct Hardness (Mpa)**	189.25 (15.29)	296.42 (12.70)	0.0051
**Ct Dissipated energy (pJ)**	1162.66 (68.10)	1279.90 (46.99)	0.0469
**Cn Indentation modulus (Gpa)**	3.72 (0.38)	6.43 (0.60)	0.0077
**Cn Hardness (Mpa)**	118.58 (10.86)	234.36 (16.61)	0.0077
**Cn Dissipated energy (pJ)**	1017.23 (39.68)	1168.98 (74.44)	NS
	**Interstitial**		
**Ct Indentation modulus (Gpa)**	8.31 (0.33)	9.98 (0.29)	0.0069
**Ct Hardness (Mpa)**	346.48 (17.99)	471.40 (12.47)	0.0051
**Ct Dissipated energy (pJ)**	1417.51 (73.22)	1673.49 (86.30)	0.0367
**Cn Indentation modulus (Gpa)**	6.82 (0.46)	9.26 (0.72)	0.0077
**Cn Hardness (Mpa)**	285.14 (17.37)	395.01 (23.30)	0.0077
**Cn Dissipated energy (pJ)**	1232.02 (73.86)	1502.68 (101.52)	0.0077

Nanoindentation parameters in cortical (Ct) and cancellous (Cn) osteonal and interstitial bone iliac crests from children with OI before and after PAM treatment (*n* = 10). Mean (SEM). Abbreviations: PAM, pamidronate; FTIRM, Fourier transform infrared microspectroscopy.

a Wilcoxon signed rank test.

**Figure 4:**
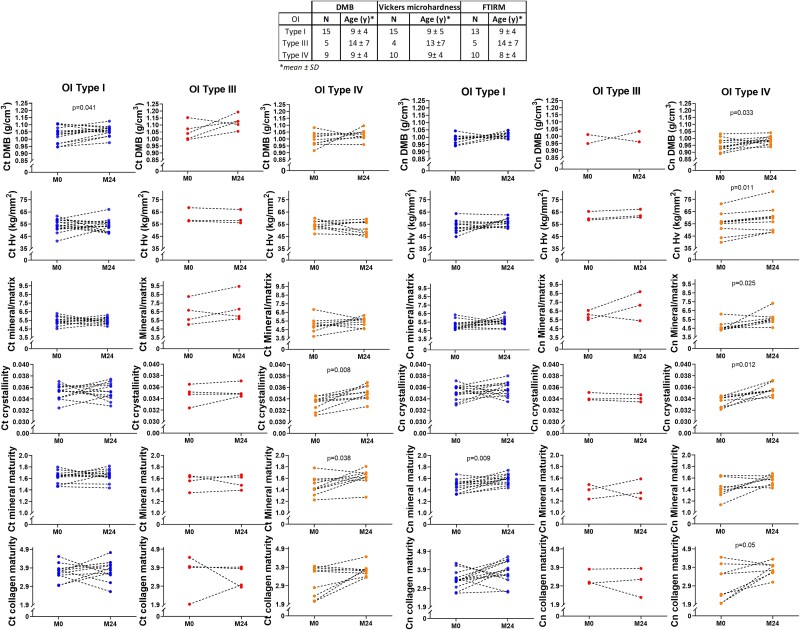
Evolution of bone material properties after 2 yr of PAM in OI type I (blue), OI type III (red), and OI type IV (orange). Abbreviations: Ct, cortical; Cn, cancellous; DMB, degree of mineralization of bone; Hv, Vickers microhardness; PAM, pamidronate; p, Wilcoxon signed rank test.

When separately analyzed by types of OI, Ct DMB was significantly increased after PAM in OI type I (*p*=.041), as well as Cn DMB in OI type IV (*p*=.033, Figure 4). No significant different was found in OI type III.

#### Mineral and organic characteristics

Mineral and organic characteristics were analyzed in 34 paired bone biopsies. Compared with baseline, 24 mo of PAM significantly increased the mineral/matrix ratio (*p*=.005) and collagen maturity (*p*=.014) in cancellous bone and the mineral maturity and crystallinity in cortical and cancellous bone .003<*p*<.043) (Table 3).

When separately analyzed by types of OI, Cn mineral/matrix ratio and Cn mineral maturity were significantly increased after PAM in OI type I (*p*=.025, *p*=.009, respectively), and Ct and Cn crystallinity and Ct mineral maturity were significantly increased after PAM in OI type IV (*p*=.008, *p*=.012 and *p*=.038, respectively, Figure 4).

#### Microhardness

Bone microhardness was analyzed in 34 paired bone biopsies. Compared with baseline, 24 mo of PAM significantly increased the microhardness of cancellous (*p*=.0009) but not of cortical bone (Table 3).

When separately analyzed by types of OI, only Cn Hv was significantly higher in OI type IV (*p*=.011, Figure 4).

#### Nanomechanical properties

Bone microhardness was analyzed in 10 paired bone biopsies. In both cortical and cancellous bone, osteonal and interstitial bone, indentation modulus, nanohardness were significantly increased after PAM treatment (.0051<*p*<.0077). Dissipated energy was increased in cortical osteonal and interstitial, cancellous interstitial (.0077<*p*<.0469) but not cancellous osteonal bone (Table 3).

**Figure 5:**
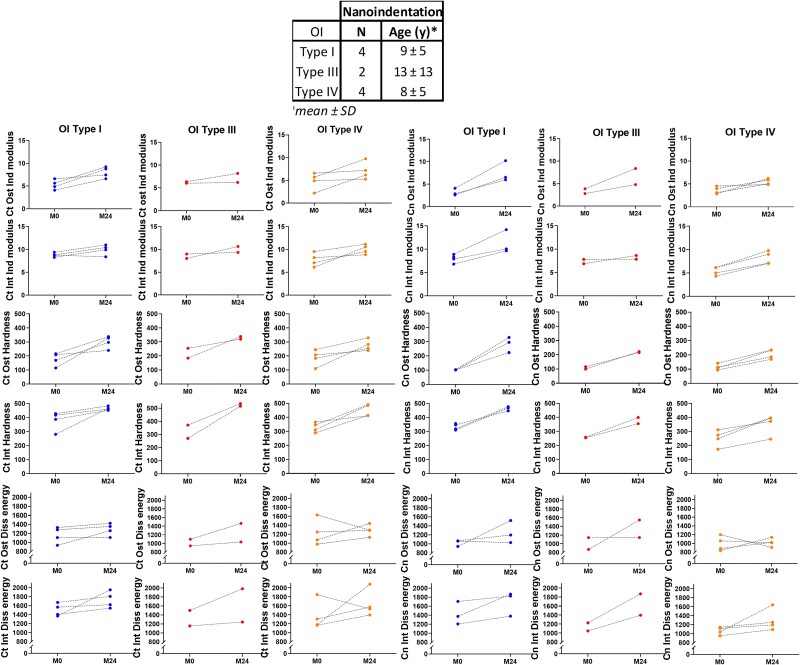
Evolution of bone nanomechanical properties properties after 2 yr of PAM in OI type I (blue), OI type III (red), and OI type IV (orange). Abbreviations: Ct, cortical; Cn, cancellous; Ost, osteonal; PAM, pamidronate; Int, interstitial; Ind modulus, indentation modulus; Diss energy, dissipated energy.

These effects were observed regardless of the type of OI (Figure 5).

## Discussion

OI is a rare disease characterized by extreme bone fragility. This bone fragility resulted not only from bone rarefaction due to reduced osteoblast activity as shown by the decreased W.Th and active formation period but also to poor matrix quality. Bone mineralization has been shown to be elevated in OI bone compared with healthy bone, regardless of the severity of the disease.^9^ This elevated mineralization was paradoxically associated with an immaturity of both collagen and mineral matrix.^31^ Thus an antiresorptive treatment such as PAM in a disease with an elevated mineralization might be questionable. Only 1 previous study has investigated the effect of PAM after 2 yr in OI children but was limited to 14 pairs of bone biopsies.^24^ The authors observed no difference in mineralization assessed by qBEI, or in nanomechanical properties after PAM in a subset of 6 pairs of bone biopsies. In this study we analyzed the effects of PAM in a higher number of bone biopsies pairs (*n* = 35) of bone biopsies from children before and after PAM using multiple complementary techniques to assess both mineral and matrix characteristics. We showed for the first time that 24 mo of PAM improved the bone nanomechanical properties associated with an increase in maturity/size of crystal mineral, and collagen maturity. After 24 mo of PAM, bone nanomechanical properties were improved, and were associated with an increase in maturity/size of crystal mineral, and collagen maturity as well. Simultaneously, bone matrix mineralization continues to increase after PAM, indicating that mineralization itself is not directly involved in the bone fragility in OI. Nevertheless, the maturation of mineral crystals, increase in size/perfection, in relationship with the increase in collagen maturity, associated with the higher bone mass, could contribute to improvement of bone strength at the organ level in patients with OI after PAM.

At baseline, we compared bone characteristics across different types of OI, but statistical analysis was only performed in OI types I, III, and IV due small sample size in other types of OI. Histomorphometric analysis revealed that Ct.Th was significantly lower in OI type IV than type I. Children in the group OI type I were 2 yr older than those in the group OI type IV, which may explain the difference in Ct.Th, as Ct.Th increases during growth.^30^ However, there is no significant correlation between the age and Ct.Th in the 3 groups OI. This suggests that the low Ct.Th in type IV is related to the disease itself rather than to the age of the children. This could contribute to the clinical severity of the disease, as OI type I is less severe than OI Type IV. However, we did not find significant lower values in OI type III, which in turn is more severe than OI Type IV, maybe due to the small sample size in the group OI type III. Mineralization was not different between the different types of OI, as previously shown.^9^ Some differences were found regarding crystallinity and mineral maturity, and nanomechanical properties with lower values in Ct Old bone from OI type IV compared with OI type I mainly. These results indicated an immaturity of mineral, with smaller crystal size/perfection in OI type IV compared with OI type I. Interestingly, OI type IV bone also showed weaker nanomechanical properties than OI type I bone, as shown by lower indentation modulus, hardness, and dissipated energy in cancellous interstitial regions.

PAM is a treatment commonly used in OI children, known to improve bone mineral density, decreasing bone turnover markers and bone pain.^23^ Histomorphometric analysis after 24 mo of PAM showed an increased cortical thickness, which reflected the modeling based-bone formation on periosteal surfaces during the growth in addition to the osteoclasts inhibition, and the marked decrease in bone turnover as previously reported.^32^ When the results were separately analyzed by type of OI, the trends of evolution of the static and dynamic parameters remained generally the same between OI types I, III, and IV. The decrease in bone turnover impacts also the mechanical properties reflected by the increased indentation modulus, hardness, and dissipated energy in both cortical and cancellous bone. Furthermore, as the local properties strongly depend on the choice of bone area,^29^ the measurements were performed at BSU level (osteonal vs interstitial) to compare the data categorized by “tissue age,” and confirmed by the systematically higher values in interstitial than in osteonal bone. The elastic modulus increases when the collagen is tested in longitudinal versus transversal direction,^33^^,^^34^ and also when the hydration decreases.^35^ In the study previously reported in 6 pairs of biopsies from OI children treated for 24 mo with PAM, no difference of nanoindentation measurements performed under dry condition was observed.^24^ This apparent discrepancy may be due to the different testing conditions. In this study, nanoindentation has been performed in wet conditions, which is closer to the physiological state. Indeed, while the bone samples have been dehydrated and embedded in MMA, some modifications of mechanical properties can be highlighted only in wet conditions, whereas they disappeared in dry conditions.^36^ As apatite crystals are surrounded by a hydrated layer highly reacting with its environment (mineral and organic matrix), the rehydration of bone with saline buffer can enhance the interaction between crystal apatite and collagen, with some differences in nanomechanical properties not observed in dry conditions as a result.

Numerous studies in human pointed out the low crystal size in OI bone mineral, assessed by different techniques such as transmission electron microscopy, X-ray diffraction (XRD) or Raman spectroscopy.^6^^,^^7^^,^^37^^,^^38^ In contrast, when assessed by small-angle X-ray scattering (SAXS) no change in crystal size (thickness) was observed,^39^ suggesting that the variations mainly concern the length.^37^ Mutations in the collagen type I encoding gene alter the triple helix structure of peptide chains and consequently the assembly of collagen network. As this collagen network serves as a template for bone mineral deposition, this strongly suggests that the altered collagen structure and/or non-collagenous protein composition, and an altered bone cell function in OI may impact the growth of mineral crystals.^1^ In addition, the bone mineral features have been shown to closely depend on the bone turnover.^40^ However, in our study we observed no significant increase in bone turnover as shown by BFR/BS, despite a higher value of Ac.f in untreated children due to a lower value of W.Th. Our results are not in agreement with Rauch’s study, which reported a significant increase in bone turnover in untreated OI children aged from 1.5 to 13.5 yr compared with healthy controls.^41^ This may be explained by the proportion of each type of OI; for example, types III and IV account for 15% and 38%, respectively, in Rauch's study, but 24% and 9% in ours.

After 24 mo of PAM, we showed for the first time that in OI the decrease in bone turnover also impacts bone matrix features, as evidenced by the increase in cortical/cancellous mineral maturity, crystallinity, and cancellous collagen maturity. Maturation of each individual crystal leads to the formation of rigid platelets, and mineral maturity reflects the low conversion of non-apatitic precursors in the hydrated layer around the crystal core into stable and stronger apatite crystals. Crystallinity, in contrast, reflects crystal size/perfection. While small crystal size in bone represents strategy to allow a degree of flexibility without fracture, the maturation toward more crystalline state increases the covalent ions inside collagen fibrils to make the bone stiffer and stronger by providing a larger energy barrier against intermolecular slip.^42^

The mean DMB of the OI bone has been assessed by X-ray microradiography. We had no iliac crests samples from healthy children to compare the DMB, but numerous studies, using different techniques, showed the high mineralization in OI independently of the clinical severity or mutations.^7–11^^,^^43^^,^^44^ While the origin for this high mineralization in OI bone is unknown, this might be related to an impaired mechanism regulating mineralization or to adaptation to compensate for the weak mechanical properties related to the low mineral maturity/small crystals size. The accumulation of numerous small crystals with an insufficient amount of collagen or an abnormal collagen impair the deformation of collagen fibrils and consequently the dissipation of the energy induced by stress. Nevertheless, the high mineralization of OI bone does not allow to compensate the low mechanical properties. Similar results have been found both in OI children and in oim mice model, that is, a lower Young’s modulus associated with an higher mineralization.^7^^,^^13^ Collagen directs the apatite crystals growth along the long-axis of the fibrils, and uniaxial ordered crystals are formed within the fibrils (intrafibrillar mineralization). In the extrafibrillar mineralization, apatite crystals are outside of the collagen fibrils and more randomly oriented.^45^ A study in dentinogenesis imperfecta type II performed by SAXS has evidenced the absence of intrafibrillar mineral.^46^ In oim mice, SAXS experiments also indicated that oim bone had deficiency of mineral in intrafibrillar space compared with WT.^21^ Thus, as the mineral/matrix is increased and intrafibrillar mineralization decreased, extrafibrillar mineralization should be increased in OI.^20^^,^^21^ Indeed, electron microscopy evidenced the presence of over mineralized regions with unorganized apatite crystals deposited onto fibril surface or in separate clusters in human OI bone.^6^ Similar observations have been made on the mineralizing oim Achilles tendon in which large crystals or aggregates of small crystals were found compared with normal animals.^47^ Therefore, it is likely that more small crystals densely packed are located outside of the collagen fibrils in OI bone. Moreover, as the mineral crystals located in extrafibrillar matrix have been shown to be more randomly oriented than those located in gap zone aligned in the longitudinal axis of collagen fibrils,^45^ this could explain the deleterious changes in load transfer between the mineral and collagen phases in OI.^21^ The intrafibrillar plasticity through mineral-collagen sliding being the predominant mechanism of toughness,^48^ this can contribute to the enhanced brittleness of OI bone. The mineralization process is a dynamic process beginning with rapid deposition of mineral corresponding to 50%-70% of the maximal value during the primary mineralization, followed by slow maturation with increasing crystals number, size/perfection, and maturity during the secondary mineralization.^40^ A recent study highlighted the accelerated kinetic of primary mineralization in type I OI compared with healthy children.^49^ In our study, MAR, which measures the rate of the primary mineralization, was slightly but significantly increased. This increase in the kinetic of the primary mineralization means that osteonal bone rapidly mineralizes, which explains the decrease in the heterogeneity of mineralization reflected by a low HI observed in our study. This absence of hypomineralized area was also observed by qBEI.^24^ A diminution of the heterogeneity of the mineralization reduces the microcracks deflection,^50^ favors their linear propagation, and consequently may contribute to the decreased energy dissipation in OI. This could also result in increased bone brittleness observed in OI.

After PAM treatment, mineralization assessed by 2 different techniques showed that cortical and cancellous DMB and cancellous mineral/matrix ratio increased. These changes reflected an accumulation of more mature crystals and an aging of organic matrix. Thus, the high mineral/matrix ratio alone cannot explain the brittleness in OI, as the ratio increased after PAM, despite a decreased fracture rate in OI children. This is rather the high mineralization, associated with the presence of immature mineral crystals, which could compromise the mechanical strength by providing a weak interaction between mineral and organic matrix. Our results are discordant with a previous study, which found no change in cancellous mineralization assessed by qBEI in 14 pairs of bone biopsies from children with OI before and after PAM.^24^ These discrepancies in results could be mainly attributed to the difference in sample size (34 pairs in our study vs 14 pairs). In our study we found an increased cancellous mineralization by 2 different techniques. X-ray microradiography can underestimate the DMB values in cancellous bone if trabecular thickness is less than 100 μm (due to partial volume effects) but not in cortical bone in which the entire cortical thickness is assessed and for which mineralization is also increased after PAM. Moreover, we also found by FTIRM an increased mineralization in cancellous bone (measured on the phosphate/organic matrix ratio on a 2 μm slice). Thus, the fact that the bone remodeling decreased after PAM, and Ct and Cn mineral maturity and crystallinity and Cn collagen maturity increased after PAM suggests an aging of bone matrix and thus an increase in mineralization is expected. Indeed, as a consequence of the marked reduction of bone turnover, antiresorptive agents prolongs the “lifespan” of the BSU, allowing a more complete secondary mineralization.^22^ Consequently, the heterogeneity of the mineralization decreases due to the larger number of well-mineralized area. In contrast, in OI, heterogeneity index of mineralization was similar after treatment. This might be due to the absence of hypomineralized bone area before treatment, confirming the elevated primary mineralization previously observed in OI.

The strength of our study lies in the comprehensive characterization of OI bone using multiple complementary techniques to assess bone remodeling, mineralization, mineral and organic features, and nanomechanical properties across a large number of OI bone biopsies both before and after PAM treatment. However, several limitations need to be mentioned. The first one is that we analyzed the effect of PAM in addition to the effect of age in growing children. The second one is that the OI histomorphometric data measured have been compared with the mean values of the age-matched normal range. Finally, a limited number of pairs for the histomorphometric analysis has been analyzed (mainly type I), due to the poor quality of some bone samples, and might not reflect the deterioration in some parameters in the more severe cases of OI.

In conclusion, compared with healthy children, OI bone is characterized by low bone mass, with a marked decrease of the amount of bone formed at the BSU level. After 24 mo, PAM decreased the bone remodeling activity that contributes to increased cortical thickness. Moreover, we showed for the first time that PAM improves bone matrix maturation, as illustrated by higher DMB, collagen/mineral maturation, and crystal size/perfection in OI children. Despite this higher mineralization after PAM, in a bone already overmineralized, the maturation of both organic and mineral matrix may promote better adhesion/cohesion forces between the crystals themselves as well as between mineral and organic phases of bone. This resulted in an improved bone matrix quality as confirmed by the increases in nanomechanical properties. Overall, the combination of bone mass gain and improvement of bone matrix quality could strongly contribute to the reduction of the fracture risk in children with OI treated with PAM.

## Supplementary Material

Supplementary_Figure_1_ziae161

Supplementary_Figure_2_ziae161

Supplementary_Figure_3_ziae161

Supplementary_captions_ziae161

## Data Availability

The data analyzed to support the findings in this study are available from the corresponding author upon request.
