# Supplementary figures and images for: Improvement of bone properties in children with osteogenesis imperfecta after pamidronate: a bone biopsy study
Source: JBMR Plus. 2024 Dec 11;9(2):ziae161. doi: 10.1093/jbmrpl/ziae161 (PMC11736749; doi:10.1093/jbmrpl/ziae161)

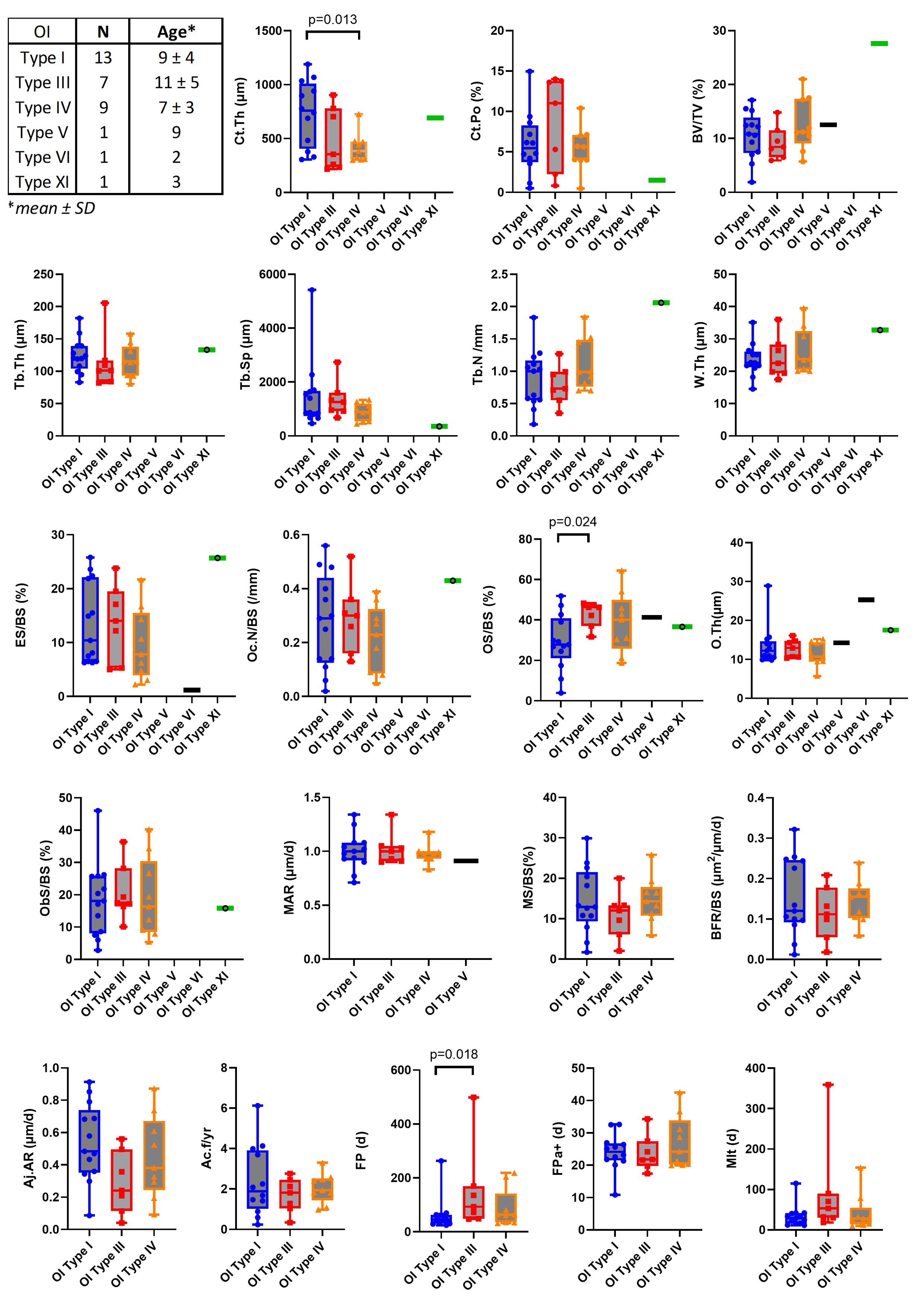

Supplement: Supplementary_Figure_1_ziae161 [file supplementary_figure_1_ziae161.jpeg]

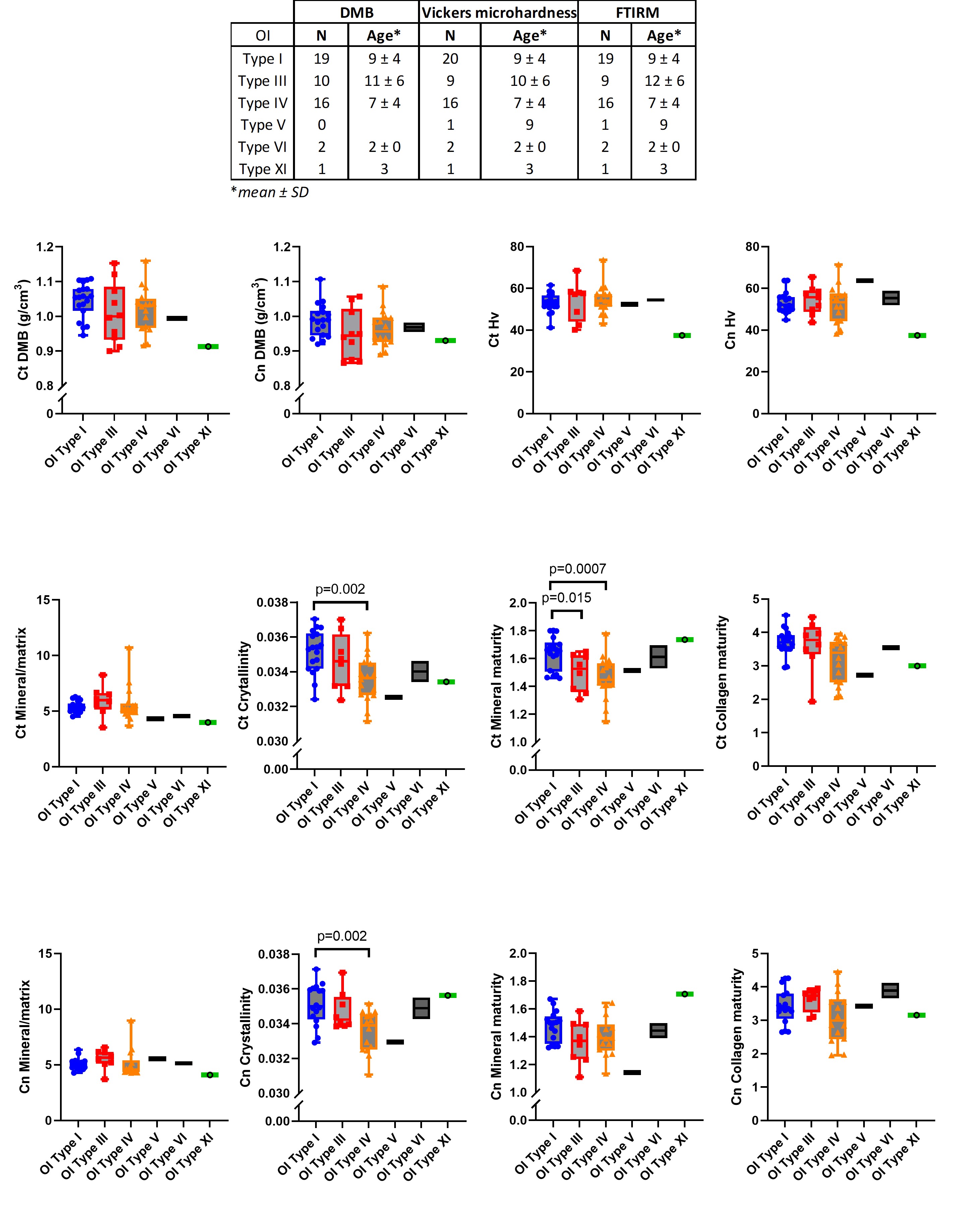

Supplement: Supplementary_Figure_2_ziae161 [file supplementary_figure_2_ziae161.jpeg]

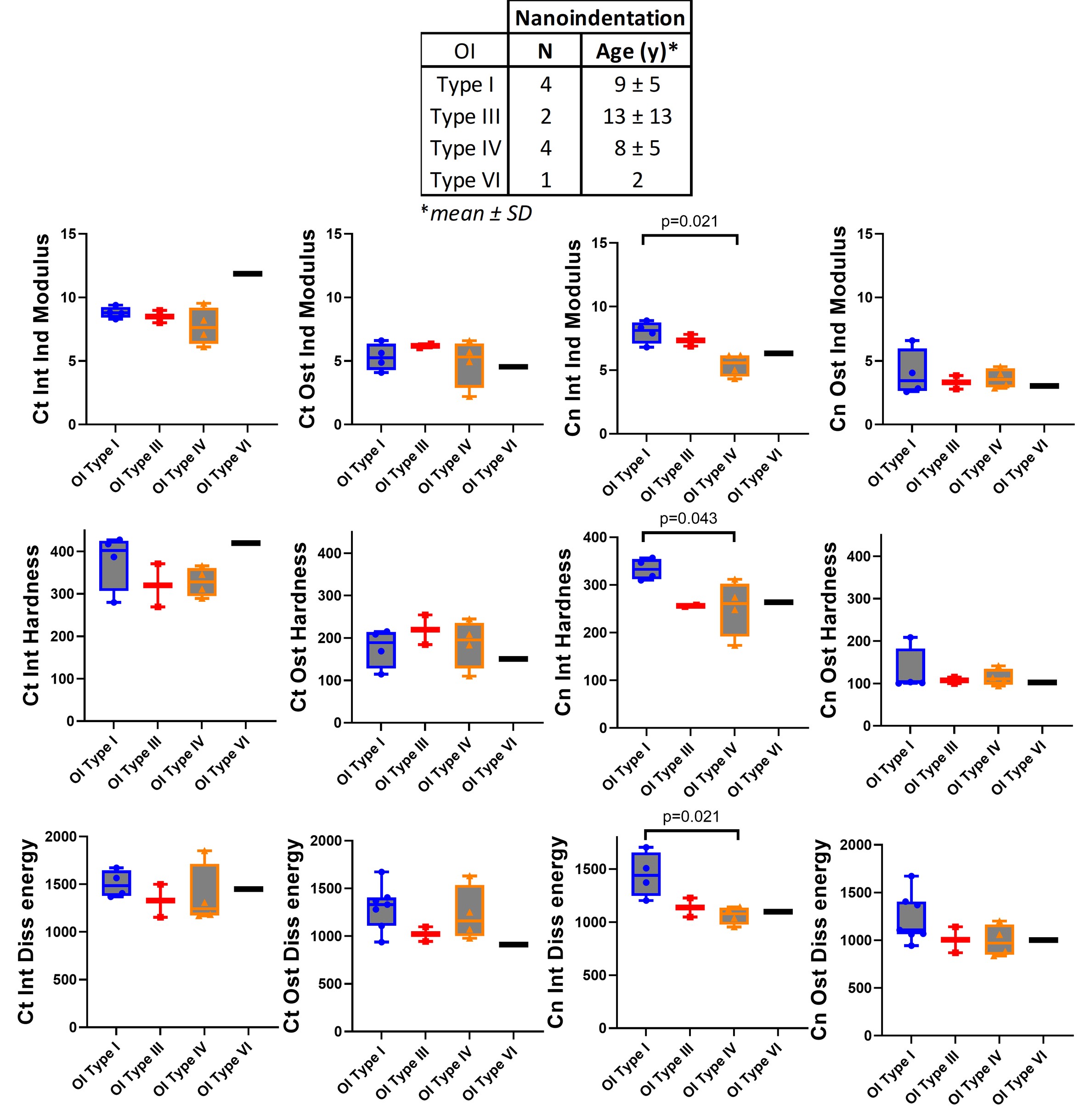

Supplement: Supplementary_Figure_3_ziae161 [file supplementary_figure_3_ziae161.jpeg]
