# Supplementary material for: Improvement of bone properties in children with osteogenesis imperfecta after pamidronate: a bone biopsy study
Source: JBMR Plus. 2024 Dec 11;9(2):ziae161. doi: 10.1093/jbmrpl/ziae161 (PMC11736749; doi:10.1093/jbmrpl/ziae161)
Supplement: Supplementary_captions_ziae161 [file supplementary_captions_ziae161.docx]

**Supplementary figure 1** Histomorphometric analysis assessed at baseline on different types of OI (OI type I, OI type III, OI type IV, OI type V, OI type VI, OI type XI). Ct: cortical; Cn: cancellous; Po: porosity; BV: bone volume; Tb: trabecular; Th: thickness; N: number; Sp: separation; W.Th: wall thickness; ES: eroded surface; Oc.S: osteoclast surface, Oc.N: osteoclast number; OS: osteoid surface; Ob.S: osteoblast surface; MAR: mineral apposition rate; MS: mineralizing surface; BFR/BS: bone formation rate; Aj.AR: adjusted apposition rate; Ac.f: activation frequency; FP: formation period; FPa+: active formation period; Mlt: mineralization lag time. p: Mann-Withney test.

**Supplementary figure 2** Bone material properties assessed at baseline on different types of OI (OI type I, OI type III, OI type IV, OI type V, OI type VI, OI type XI). Ct: cortical; Cn: cancellous; DMB: Degree of mineralization of bone; Hv: Vickers microhardness, Fourier Transform Infrared analysis (mineral/matrix ratio, crystallinity, mineral maturity and collagen maturity); p: Mann-Withney test.

**Supplementary figure 3** Bone nanomechanical properties assessed at baseline on different types of OI (OI type I, OI type III, OI type IV and OI Type VI). Ct: cortical; Cn: cancellous; Ost: osteonal; Int: interstitial, Ind modulus: Indentation modulus; Diss energy: Dissipated energy. p: Mann-Withney test.
